# The Association of the *GABRP* Polymorphisms with Systemic Lupus Erythematosus

**DOI:** 10.1155/2015/602154

**Published:** 2015-11-08

**Authors:** Hun-Soo Kim, Eun-Heui Jin, Ji-Su Mo, Hyeok Shim, Shin-Seok Lee, Soo-Cheon Chae

**Affiliations:** ^1^Department of Pathology, School of Medicine, Wonkwang University, Iksan, Chonbuk 570-749, Republic of Korea; ^2^Department of Internal Medicine, School of Medicine, Wonkwang University, Iksan, Chonbuk 570-749, Republic of Korea; ^3^Division of Rheumatology, Department of Internal Medicine, Chonnam National University Medical School, Gwangju 501-746, Republic of Korea

## Abstract

Gamma-aminobutyric acid receptor subunit pi (GABRP) is involved in inhibitory synaptic transmission in the central nervous system. This gene encodes multisubunit chloride channels and is also expressed in numerous nonneuronal tissues such as the uterus and the ovaries. This study was aimed to validate whether the polymorphisms in the GABRP gene are associated with the susceptibility to systematic lupus erythematosus (SLE). The genotype frequencies of the rs929763, rs732157, and rs3805455 of the *GABRP* gene in SLE patients were significantly different from those of the control group (*P* < 0.0001, *P* = 0.05 and 0.002, resp.). Additional analysis showed that the genotype of the rs929763 and rs3805455 of the *GABRP* gene were also significantly associated with female SLE patients (*P* < 0.0001, *P* = 0.005, resp.). Two haplotype frequencies including a major haplotype of *GABRP* SNPs were more significantly different between the SLE patients and the healthy controls (*P* = 0.038 and 4.2*E* − 24, resp.). These results suggest that the polymorphisms in the *GABRP* gene might be associated with the susceptibility to SLE and the haplotype of *GABRP* SNPs is useful genetic marker for SLE.

## 1. Introduction

Systemic lupus erythematosus (SLE) is a chronic autoimmune disease that can affect almost any organ system and has erratic manifestations and follows a relapsing and remitting course. More than 90% of cases of SLE occur in women, frequently starting at childbearing age. The exact cause of SLE is not known, but several factors have been associated with the disease including genetic, environmental, and hormonal factors [1, 2]. Although SLE is not linked to a certain gene, the people with SLE often have members of their family with other autoimmune conditions. The treatment for SLE is not curative but mainly focused on lessening the symptoms and reducing its long term complications. Treatment options can vary depending on the severity and the location of the body or organ that are affected. These include corticosteroids, immunosuppressants, or antimalarial drugs.

We previously made a customized 3K SNPs chip containing the presumable SNPs associated with various immune disorder, such as rheumatic arthritis (RA) and SLE, and carried out a pilot study using the genomic DNA samples of RA and SLE patients. We identified 16 candidate genes, including the gamma-aminobutyric acid receptor pi (*GABRP*), epithelial stromal interaction 1 (*EPSTI1*), bone morphogenetic protein 6 (*BMP6*), integrin beta 5 (*ITGB5*), spectrin repeat containing nuclear envelope 1 (*SYNE1*), and TIMP metallopeptidase inhibitor 3 (*TIMP3*), from SLE (Our not published data). These results led us to determine whether the SNPs of the* GABRP* gene are associated with the susceptibility of SLE.

The gamma-aminobutyric acid (GABA), the major inhibitory neurotransmitter in the brain, mediates neuronal inhibition by binding GABRP, a multisubunit chloride channel, and opening an integral chloride channel. Transcripts of this subunit could be detected in several human tissues, especially in the uterus, in which the function of the receptor appears to be related to tissue contractility [3, 4]. Variations at the* GABA* receptor gene family are associated with susceptibility to neuropsychiatric bipolar schizoaffective disorder [5, 6]. The expression levels of* GABRP* genes are relatively high in normal and benign human epithelial cells of the breast compared to other normal tissues and even compared with neuronal tissues of the CNS. There is progressive downregulation in concordance with tumor progression in sporadic breast cancer tissues [7]. Expression of GABRP is also associated with the basal-like/triple negative subtype, brain metastases and poorer prognosis [8].

In this study, we analyzed the allelic and genotypic frequencies of* GABRP* SNPs between the SLE patients and the healthy controls. Furthermore, we investigated haplotype frequencies constructed by these SNPs in both groups.

## 2. Materials and Methods

### 2.1. Patients and DNA Samples

The DNA samples used in this study were provided by the Biobank of Wonkwang University Hospital, a member of the National Biobank of Korea, which is supported by the Ministry of Health and Welfare Affairs. On the basis of approval and informed consent from the institutional review board, we obtained the genomic DNAs from 164 SLE patients (12 males and 152 females) and 528 healthy controls (329 males and 199 females). Mean ages of SLE patients and healthy controls were 40.6 years and 34.4 years, respectively. Genomic DNA was extracted from peripheral blood leukocytes by using a standard phenol-chloroform method or by using a Genomic DNA Extraction kit (iNtRON Biotechnology, Korea) according to the manufacturer's directions. SLE patients were recruited from the outpatient clinic at Chonnam National University Hospital. SLE was diagnosed according to criteria of the American College of Rheumatology (ACR) [9]. Antinuclear antibody (ANA) levels in SLE patients were determined in a routine laboratory at Chonnam National University Hospital. The controls were recruited from the general population and had received comprehensive medical testing at the Wonkwang University Hospital.

### 2.2. SNP Selection and Genotype Analysis

We selected six SNPs (rs929763 [T/A, intron 1], rs732157 [C/T, intron 1], rs2303134 [Asn55Ser, A/G, exon 3], rs1063310 [Phe391Len, C/A, exon 10], rs3805455 [C/T, 3′-UTR], and rs3828619 [G/A, 3′-UTR]) of the* GABRP* based on their location, minor allele frequencies (MAF < 0.05), and linkage disequilibrium (LD) analysis from NCBI SNP database. Genotyping was performed by high resolution melting (HRM) analysis. The 10 *μ*L reaction mixture was made up using 1x QuantiTect Probe PCR Kit (Qiagen, USA) and consisted of 50 ng of genomic DNA, 100 nM of each primer (Table 1), and 1x Evagreen solution (Biotium, USA). PCR cycling and HRM analysis were carried out using the Rotor-Gene thermal cycler RG6000 (Corbett Research, Australia). The PCR was performed in the following conditions: one cycle at 95°C for 15 min and 45 cycles of 95°C for 15 sec, 68°C for 10 sec, and 72°C for 30 sec. Optical measurements in the green channel (excitation at 470 nm and detection at 519 nm) were recorded during the extension step. After completion of 45 cycles, melting-curve data were generated by increasing the temperature from 77 to 95°C at 0.1°C per second and recording fluorescence. HRM curve analysis was performed using the software Rotor-Gene 1.7.40 and the HRM algorithm provided.

### 2.3. Statistical Analysis

SLE patients and healthy control groups were compared using case-control association analysis. The *χ* ^2^ test was used to estimate Hardy-Weinberg equilibrium (HWE). Allele frequency was defined as the percentage of individuals carrying the allele among the total number of individuals. Logistic regression analyses (SPSS 11.5) were used to calculate odds ratios (95% confidence interval) for SNP sites. Linkage disequilibrium (LD) analyses by pair-wise comparison of biallelic loci and haplotype frequencies of the* GABRP* gene for multiple loci were estimated using the expectation maximization (EM) algorithm with SNPAlyze software (DYNACOM, Yokohama, Japan). The ANOVA was applied to analyze differences between the genotype and ANA levels in SLE patients. A *P* value of less than 0.05 was considered an indication of statistical significance.

## 3. Results

The human* GABRP* gene is located on chromosome 5q35.1 and consists of 10 (NM_014211.2, isoform 1) or 9 exons (NM_001291985.1, isoform 2). The* GABRP* gene was identified as a candidate gene associated to SLE by our previous pilot study using customized 3K SNPs chip (our data not published).

To determine whether the six* GABRP* SNPs are associated with SLE susceptibility, the genotypes of the* GABRP* polymorphisms were analyzed by HRM method, and the genotype and allelic frequencies between the both groups were compared. All the genotype frequencies in both the healthy controls and the SLE patients were consistent with HWE, except for rs3828619 in SLE (data not shown). The genotype frequencies in four of the six* GABRP* SNPs, rs929763, rs732157, rs3805455, and rs3828619, in the SLE group were significantly different from the healthy control group (*P* < 0.0001, *P* = 0.05, 0.002, and 0.003, resp.; Table 2). The allele frequencies of* GABRP* SNPs, rs929763, rs3805455, and rs3828619, in the SLE group also were significantly different from those in the healthy control group (*P* = 0.0005, 0.04, and 0.005, resp.; Table 2). These results strongly suggest that the SNPs of* GABRP* appear to be associated with SLE susceptibility. Since SLE, like many other autoimmune diseases, affects females more frequently than males, at a rate of almost 9 to 1 [10], we independently analyzed the 152 female patients (92.7%) out of the total of 164 SLE patients recruited in the study, in order to find out if gender has an important role in the high susceptibility of the* GABRP* SNPs and SLE. The genotype frequencies of the rs929763, rs3805455, and rs3828619 in female SLE patients were statistically different from those of female healthy controls (*P* < 0.0001, *P* = 0.005 and 0.008, resp.; Table 3).

We further estimated the haplotype frequencies of SNPs (rs929763, rs732157, rs2303134, rs1063310, and rs3805455) of the* GABRP* gene between healthy controls and SLE patients (Table 4). Out of 32 possible haplotypes, three haplotypes (TCACC, ACACC, and ATAAT) were identified as the main haplotypes (>5%) in both groups (Table 4). The distribution of the major haplotype (TCACC) was significantly different in the SLE patients compared to that of the healthy controls (*P* = 0.038). Interestingly, the distribution frequency of the ACACT haplotype in SLE patients group was hugely different than that in healthy controls group (*P* = 4.2*E* − 24; Table 4). This ACACT haplotype is distributed very infrequently in healthy control groups (0.064%), compared to it being one of the main haplotype in the SLE patient group (10.5%). This haplotype frequency difference of TCACC and ACACT in both groups is especially true when only the female subjects were analyzed (*P* = 7.7*E* − 4 and 2.7*E* − 11, resp.; Table 5). These results suggest that* GABRP* polymorphisms might be an important genetic factor associated with SLE susceptibility.

Finally, to define a possible correlation between* GABRP* polymorphisms and the clinical features of SLE, we further analyzed the difference of the antinuclear antibody (ANA) levels according to each genotype of the SLE patients. We found that these SNPs in the SLE patients have no significant association with the levels of ANA (Table 6).

## 4. Discussion

SLE is an autoimmune disorder with autoantibody-mediated tissue damage. SLE is clinically characterized by heterogeneous symptoms which involves almost all organs in the body. Management of this disease is complex and usually involves many different specialties for optimal patient management [11]. We previously reported that SNPs in the forkhead-box J1 (*FOXJ1*), interleukin coactosin-like 1 (*COTL1*), and thymic stromal lymphopoietin receptor (*TSLPR*) genes are associated with susceptibility to SLE in a Korean population [12–14]. In this study, we evaluated the association between* GABRP* polymorphisms and susceptibility to SLE.

We previously identified the several candidate genes including* GABRP* gene associated with SLE by our pilot study using customized 3K SNPs chip (our data not published). The mRNA expression levels of GABRP in 23 normal human tissues were diagrammatically presented in a study done by Zafrakas et al. [7]. Five out of the 23 normal tissues were directly or indirectly related to human lymphoreticular and immune system (lymph node, thymus, bone marrow, spleen, and liver). Among them, none of them showed any significant increase in* GABRP* mRNA expression. The main physiologic function of GABRP in nonneuronal tissue and its role in disease is not well known. Only a few manuscripts have been acknowledged for having association, and these are mostly limited to breast cancer and neuropsychiatric disorders. Our putative data has suggested that the polymorphism of the human* GABRP* gene is strongly associated with the susceptibility to SLE (Tables 2 and 3). It could be promptly argued that the expression levels of GABRP and its involvement in the immune system could be difficult to connect. Only a single study was published evaluating the relationship between acute and chronic rejections of renal allografts to 345 genes that provided potential relevance to renal allograft rejection. This study demonstrated that there was increased expression of GABRP along with 8 other genes in the grafts that failed [15].

We demonstrated that the genotype frequencies of* GABRP* polymorphisms (rs929763, rs732157, and rs3805455) in SLE patients were significantly different from that of the healthy control group to such a degree that defies any possibility of random chance (Table 2). Specifically the genotype and allele frequencies of rs929763 had very high associations (*P* < 0.0001 and 0.0005, resp.). This strong association is also true when the subjects are confined to the female population (Table 3). These results confidently led us to think that* GABRP* gene polymorphism have a strong influence on the susceptibility to SLE.

The distributions of the major haplotypes (TCACC) of the* GABRP* SNPs, rs929763, rs732157, rs2303134, rs1063310, and rs3805455, in the SLE patients were significantly different from that of the healthy controls (*P* = 0.038; Tables 4 and 5). The frequency of other haplotype (ACACT) of the* GABRP* SNPs was more profoundly different between both groups (*P* = 4.2*E* − 24). These results suggest that* GABRP* polymorphisms might be an important genetic factor associated with SLE susceptibility.

We also compared ANA levels among the genotypes of polymorphisms of* GABRP* gene in SLE (Table 6). The ANA test detects the autoantibodies present in an individual's blood serum. ANA titers are useful in diagnosis of various autoimmune disorders including SLE and monitoring levels help to predict the progression of disease [16, 17]. We could not find any correlation with ANA level between the genotypes of* GABRP* polymorphism, suggesting that the* GABRP* polymorphism is only linked to SLE susceptibility and is not associated with disease progression.

In conclusion, the results of this study strongly suggest that the* GABRP* gene might be a candidate gene associated with the susceptibility of SLE, and our result also indicates that the haplotypes of the* GABRP* polymorphisms might be one of the influential genetic markers for SLE susceptibility. Although it is not clear how the* GABRP* polymorphisms are related to the pathogenesis of SLE, our results could provide valuable resource for further functional studies of the* GABRP* gene and its relationship with other various autoimmune or inflammatory disorders. Actually* GABRP* gene is located in 5q3 locus.

## Figures and Tables

**Table 1:** Primer sequences used for genotyping of *GABRP *gene.

Primer	Sequence (5′ → 3′)	SNP
GABRP-F1	TCCAGGTGTCTGGAGAGAGG	rs929763
GABRP-R1	CAGCAGGGGCTCTAATCTTG	

GABRP-F2	TGGCAATAATGCCTTTCCTC	rs732157
GABRP-R2	TTGTCACCTCCAGCTTCCTT	

GABRP-F3	CCTGGCTTTGAGAACCTCAC	rs2303134
GABRP-R3	TAAAACCTCCCGAAACCCTC	

GABRP-F4	AGATCAGCTTTGCCAGCATT	rs1063310
GABRP-R4	CAACAATCCTGCCCATCTTT	

GABRP-F5	GCTGGCCCTGAGTACTGAAC	rs3805455
GABRP-R5	CTCTGGCTGGATTTGGAGAG	

GABRP-F6	GAGCCACAGGTTCTCATTCC	rs3828619
GABRP-R6	CCTCTTTTCACCAGCACTCC	

**Table 2:** Genotype and allele analyses of the *GABRP *gene SNPs in SLE patients and healthy controls.

Position^a^	Genotype/allele	Control *n* (%)	SLE *n* (%)	Odds ratio^b^ (95% CI)	*P* ^c^
rs929763	TT	260 (50.4)	68 (42.5)	1.00	**<0.0001**
	TA	222 (43.0)	61 (38.1)	1.05 (0.71–1.55)	
	AA	34 (6.6)	31 (19.4)	3.49 (2.00–6.07)	
	T	742 (71.9)	197 (61.6)	1.00	**0.0005**
	A	290 (28.1)	123 (38.4)	1.60 (1.23–2.08)	

rs732157	CC	261 (50.5)	96 (59.3)	1.00	**0.05**
	CT	218 (42.2)	57 (35.2)	0.71 (0.49–1.03)	
	TT	38 (7.4)	9 (5.5)	0.64 (0.30–1.38)	
	C	740 (71.6)	249 (76.9)	1.00	0.06
	T	294 (28.4)	75 (23.1)	0.76 (0.57–1.02)	

rs2303134	AA	518 (98.1)	162 (98.8)	1.00	0.57
	AG	10 (1.9)	2 (1.2)	0.64 (0.14–2.95)	
	GG	0 (0.0)	0 (0)	—	
	A	1046 (99.1)	326 (99.4)	1.00	0.57
	G	10 (0.9)	2 (0.6)	0.64 (0.14–2.94)	

rs1063310	CC	233 (45.0)	82 (50.9)	1.00	0.18
	CA	237 (45.8)	66 (41.0)	0.79 (0.55–1.15)	
	AA	48 (9.3)	13 (8.1)	0.77 (0.40–1.49)	
	C	703 (67.9)	230 (71.4)	1.00	0.23
	A	333 (32.1)	92 (28.6)	0.84 (0.64–1.11)	

rs3805455	CC	226 (43.9)	63 (40.4)	1.00	**0.002**
	CT	243 (47.2)	65 (41.7)	0.96 (0.65–1.42)	
	TT	46 (8.9)	28 (17.9)	2.18 (1.26–3.77)	
	C	695 (67.5)	191 (61.2)	1.00	**0.04**
	T	335 (32.5)	121 (38.8)	1.31 (1.01–1.71)	

rs3828619	GG	299 (57.2)	73 (48.7)	1.00	**0.003**
	GA	181 (34.6)	53 (35.3)	1.20 (0.80–1.79)	
	AA	43 (8.2)	24 (16.0)	2.29 (1.30–4.01)	
	G	779 (74.5)	199 (66.3)	1.00	**0.005**
	A	267 (25.5)	101 (33.7)	1.48 (1.12–1.95)	

^a^Calculated from the translation start site.

^b^Logistic regression analyses were used for calculating OR (95% CI; confidence interval).

^c^Value was determined by Fisher's exact test or *χ* ^2^ test from a 2 × 2 contingency table.

**Table 3:** Genotype and allele analyses of the *GABRP* gene polymorphisms in the female SLE patients and female healthy controls.

Position^a^	Genotype/allele	Control *n* (%)	SLE *n* (%)	Odds ratio^b^ (95% CI)	*P* ^c^
rs929763	TT	109 (56.8)	63 (42.6)	1.00	**<0.0001**
	TA	73 (38.0)	56 (37.8)	1.33 (0.83–2.12)	
	AA	10 (5.2)	29 (19.6)	5.02 (2.29–10.98)	
	T	291 (75.8)	182 (61.5)	1.00	**<0.0001**
	A	93 (24.2)	114 (38.5)	1.96 (1.410–2.73)	

rs732157	CC	108 (54.8)	89 (59.3)	1.00	0.40
	CT	76 (38.6)	52 (34.7)	0.83 (0.53–1.30)	
	TT	13 (6.6)	9 (6.0)	0.84 (0.34–2.06)	
	C	292 (74.1)	230 (76.7)	1.00	0.44
	T	102 (25.9)	70 (23.3)	0.87 (0.61–1.24)	

rs2303134	AA	193 (97.0)	150 (98.7)	1.00	0.29
	AG	6 (3.0)	2 (1.3)	0.43 (0.09–2.16)	
	GG	0 (0)	0 (0)	—	
	A	392 (98.5)	302 (99.3)	1.00	0.29
	G	6 (1.5)	2 (0.7)	0.43 (0.09–2.16)	

rs1063310	CC	86 (43.9)	77 (51.7)	1.00	0.1
	CA	95 (48.5)	59 (39.6)	0.69 (0.44–1.09)	
	AA	15 (7.6)	13 (8.7)	0.97 (0.43–2.16)	
	C	267 (68.1)	213 (71.5)	1.00	0.34
	A	125 (31.9)	85 (28.5)	0.85 (0.61–1.19)	

rs3805455	CC	89 (44.9)	60 (41.7)	1.00	**0.005**
	CT	93 (47.0)	58 (40.3)	0.93 (0.58–1.47)	
	TT	16 (8.1)	26 (18.1)	2.41 (1.19–4.87)	
	C	271 (68.4)	178 (61.8)	1.00	**0.07**
	T	125 (31.6)	110 (38.2)	1.34 (0.97–1.84)	

rs3828619	GG	113 (57.4)	68 (48.9)	1.00	**0.008**
	GA	71 (36.0)	50 (36.0)	1.17 (0.73–1.87)	
	AA	13 (6.6)	21 (15.1)	2.68 (1.26–5.71)	
	G	297 (75.4)	186 (66.9)	1.00	**0.02**
	A	97 (24.6)	92 (33.1)	1.51 (1.08–2.13)	

^a^Calculated from the translation start site.

^b^Logistic regression analyses were used for calculating OR (95% CI; confidence interval).

^c^Value was determined by Fisher's exact test or *χ* ^2^ test from a 2 × 2 contingency table.

**Table 4:** Haplotype frequencies of *GABRP *SNPs in SLE patients and healthy controls.

Haplotype					Frequency^a^		Chi-square	*P* ^b^
rs929763	rs732157	rs2303134	rs1063310	rs3805455	Control	SLE		
T	C	A	C	C	0.406	0.345	3.580	0.03
A	C	A	C	C	0.228	0.220	0.091	0.803
A	T	A	A	T	0.223	0.185	1.942	0.166
A	C	A	A	T	0.037	0.057	2.516	0.334
T	C	A	A	T	0.044	0.026	1.893	0.431
A	C	A	C	T	6.4*E* − 4	0.105	102.6	4.2**E** − 24
Other					0.062	0.062	—	—

^a^Values were constructed by EM algorithm with genotyped SNPs.

^b^Values were analyzed by permutation test or Chi-square.

**Table 5:** Haplotype frequencies of *GABRP *SNPs in female SLE patients and female healthy controls.

Haplotype					Frequency^a^		Chi-square	*P* ^b^
rs929763	rs732157	rs2303134	rs1063310	rs3805455	Control	SLE		
T	C	A	C	C	0.645	0.514	11.31	7.7**E** − 4
A	T	A	A	T	0.195	0.186	0.099	0.753
T	C	A	A	T	0.097	0.070	0.585	0.444
A	C	A	C	T	3.2*E* − 30	0.113	44.36	2.7**E** − 11
A	C	A	C	C	0.013	0.043	5.917	0.015
Other					0.050	0.074	—	—

^a^Values were constructed by EM algorithm with genotyped SNPs.

^b^Values were analyzed by permutation test or Chi-square.

**Table 6:** The levels of ANA among the genotypes of polymorphisms of *GABRP* gene in SLE patients.

SNP	Genotype	ANA			*P* ^a^
		*N*	Mean	SD	
rs929763	TT	41	577.1	1.7*E* + 3	0.720
	TA	44	874.6	3.1*E* + 3	
	AA	27	471.1	6.3*E* + 2	

rs732157	CC	67	510.7	1.3*E* + 3	0.478
	CT	40	986.0	3.3*E* + 3	
	TT	7	205.7	2.0*E* + 2	

rs2303134	AA	115	652.5	2.2*E* + 3	0.880
	AG	1	320.0	—	
	GG	0	—	—	

rs1063310	CC	56	559.6	1.4*E* + 3	0.731
	CA	49	834.3	3.0*E* + 3	
	AA	8	300.0	2.3*E* + 2	

rs3805455	CC	39	621.0	1.7*E* + 3	0.684
	CT	47	863.0	3.1*E* + 3	
	TT	23	372.2	5.0*E* + 2	

rs3828619	GG	48	536.3	1.5*E* + 3	0.521
	GA	38	1031.6	3.4*E* + 3	
	AA	19	425.3	5.4*E* + 2	

^a^Values were analyzed by ANOVA.
